# Supplementary material for: Conditional chemoconnectomics (cCCTomics) as a strategy for efficient and conditional targeting of chemical transmission
Source: eLife. 2024 Apr 30;12:RP91927. doi: 10.7554/eLife.91927 (PMC11060718; doi:10.7554/eLife.91927)
Supplement: Figure 3—source data 3. [file elife-91927-fig3-data3.zip › Figure 3 source data3 part 1.zip!/Figure 3-Source data 3-part1/1082-9-CRISPResso_Report_zod2ewbs.zip!/CRISPResso_on_1082_10/2a.Nucleotide_percentage_quilt.pdf]

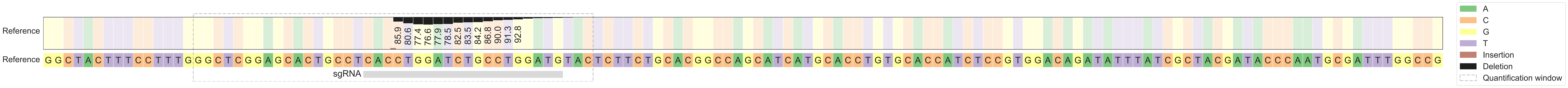
Reference
85.9
80.6
77.4
76.6
77.9
78.5
82.5
83.5
84.2
86.8
90.0
91.3
92.8
Reference
GGCTACTTTCCTTTGGGCTCGGAGCACTGCCTCACCTGGATCTGCCTGGATGTACTCTTCTGCACGGCCAGCATCATGCACCTGTGCACCATCTCCGTGGACAGATATTTATCGCTACGATACCCAATGCGATTTGGCCG
sgRNA
A
C
G
T
Insertion
Deletion
Quantification window
